# SARS-CoV-2 Infection in Pregnant Women With Hypothyroidism

**DOI:** 10.7759/cureus.61206

**Published:** 2024-05-27

**Authors:** Madalina Daniela Iordache, Daniela Catalina Meca, Monica Mihaela Cirstoiu

**Affiliations:** 1 Department of Obstetrics and Gynaecology, University Emergency Hospital Bucharest, Carol Davila University of Medicine and Pharmacy, Bucharest, ROU

**Keywords:** blood count, 1-min apgar score, preterm birth, low birth weight, hypothyroidism, sars-cov-2 infection, pregnancy

## Abstract

Background

Severe acute respiratory syndrome coronavirus 2 (SARS‑CoV‑2) infection has been linked to increased maternal and fetal morbidity and mortality, as evidenced by numerous studies. Given the potential exacerbation of autoimmune diseases during viral infections, maternal and fetal complications such as preterm birth, low birth weight, or preeclampsia, often observed in pregnancies involving autoimmune thyroiditis with hypothyroidism, may be further aggravated. This study seeks to ascertain whether the association between viral infection and hypothyroidism contributes to an increase in adverse pregnancy outcomes.

Methods

This study included a cohort of 145 pregnant women with SARS-CoV-2 infection, who delivered in the Department of Obstetrics and Gynecology of the University Emergency Hospital in Bucharest, Romania, between January 1, 2020, and December 31, 2022. The participants were divided into two groups depending on the presence of autoimmune thyroiditis with hypothyroidism. We examined the maternal and fetal demographic parameters, paraclinical laboratory parameters, and outcomes, aiming to identify disparities between the two groups.

Results

Among the 145 SARS-CoV-2-positive pregnant women, the prevalence of hypothyroidism was 8.96%, with 13 cases reported. In the hypothyroidism group, the mean age of coronavirus disease 2019 (COVID-19) patients was higher (34.07 ± 5.18 years vs. 29.25 ± 6.23 years), as was the number of cases of investigated pregnancies, 12 (92.31%) vs. 91 (68.94%). There was no statistically significant correlation observed between fetal weight at birth, one-minute Apgar score, neonatal intensive care unit (NICU) admission, or intrauterine growth restriction between the two groups. Nevertheless, a case of stillbirth was recorded in the hypothyroidism group. The presence of thyroid pathology did not exacerbate the progression of the viral infection, as evidenced by the absence of cases of preeclampsia, ICU admission, or SARS-CoV-2 pneumonia. Conversely, the presence of hypothyroidism in pregnant women with SARS-CoV-2 infection was associated with lower uric acid levels and a slight decrease in international normalised ratio (INR) values. Additionally, there was a significant negative association between uric acid levels and the one-minute Apgar score in the hypothyroidism group, while no such correlations were observed in the other group. Furthermore, there was a statistically significant correlation between intrauterine growth restriction and uric acid values, as well as between the one-minute Apgar score and INR parameters, in both groups.

Conclusion

The link between SARS-CoV-2 infection and hypothyroidism does not appear to increase the risk of preterm birth, intrauterine growth restriction, or low fetal weight at birth. However, it may be associated with a higher risk of stillbirth. The presence of hypothyroidism in pregnant women with COVID-19 correlates with lower maternal uric acid levels and a slight decrease in INR values. The one-minute Apgar score correlates with the level of uric acid in pregnant women with SARS-CoV-2 infection and hypothyroidism.

## Introduction

The physiological alterations in the immune and cardiorespiratory systems during pregnancy, such as the shift towards a Th2-dominant immune response, increased oxygen consumption, and decreased functional residual capacity, result in a modified reaction to severe acute respiratory syndrome coronavirus 2 (SARS‑CoV‑2) infection [1,2]. Despite pregnant women having a comparable incidence rate to non-pregnant women, coronavirus disease 2019 (COVID-19) is linked to heightened disease severity among pregnant individuals, including a higher likelihood of ICU admission or maternal death, as well as an elevated risk of iatrogenic preterm birth [3-7].

Infection with SARS-CoV-2 exhibits a bi-directional effect: it can lead to the production of autoantibodies during clinical disease even without a pre-existing autoimmune condition, and it can exacerbate the clinical course of autoimmune diseases due to the viral infection [8,9].

Autoimmune thyroiditis with hypothyroidism has a prevalence of 2-3% during pregnancy [10]. A study conducted by Atalay et al. in 2023, investigating the effect of COVID-19 on thyroid function tests and considering that the severity of SARS-CoV-2 infection amplifies perinatal complications, proposed that free triiodothyronine (FT3) levels are associated with the severity of the viral infection [11].

Untreated hypothyroidism results in preterm birth, low birth weight, and maternal complications like preeclampsia and postpartum hemorrhage [12]. Conversely, symptomatic COVID-19 during pregnancy is linked to preterm birth and a heightened likelihood of cesarean delivery [2]. Despite the increase in maternal mortality and morbidity due to SARS-CoV-2 infection, it does not elevate the risk of stillbirth or intrauterine growth restriction [13]. On the other hand, maternal hypothyroidism may serve as a predisposing factor for neonatal SARS-CoV-2 transmission, considering the potential impact of thyroid dysfunction on the placental barrier [14]. Although the treatment of hypothyroidism in pregnant women with COVID-19 is similar to that in the non-pregnant population, the management should be individualized [15].

## Materials and methods

This was a descriptive, retrospective study involving 145 pregnant women who tested positive for SARS-CoV-2 and delivered in the Department of Obstetrics and Gynecology of University Emergency Hospital in Bucharest, Romania, between January 1, 2020, and December 31, 2022. The study was approved by the Ethical Committee of the University Emergency Hospital in Bucharest (approval number: 58134/16.11.2020). Maternal and fetal clinical and paraclinical data were retrieved from the Data Base System of the University Emergency Hospital in Bucharest and observation sheets. Informed consent was obtained from all the participants in the study.

The inclusion criteria comprised all pregnant women over 18 years of age with a positive reverse transcriptase-polymerase chain reaction (RT-PCR) test for COVID-19 admitted to the University Emergency Hospital in Bucharest, and the presence of informed consent. The exclusion criteria included all patients who had COVID-19 during pregnancy but tested negative at admission, multiple pregnancies, and pregnancies following assisted reproductive technology.

The participants were categorized into two groups based on the presence of autoimmune thyroiditis with hypothyroidism. In the hypothyroidism group, we included all patients already diagnosed with this condition, who had undergone thyroid stimulating hormone (TSH) and free thyroxine (fT4) blood tests upon hospital admission. All the patients included had a value of anti-thyroid peroxidase (anti-TPO) over 100 IU/mL. The exclusion criteria in the hypothyroidism group included the absence of thyroid function tests (TSH, fT4) or the absence of RT-PCR for SARS-CoV-2.

Data analysis was performed using IBM SPSS Statistics for Windows, Version 26.0 (Released 2019; IBM Corp., Armonk, New York, United States). Pearson's chi-square test was applied when dealing with categorical outcomes, regardless of the number of categories in the outcomes or exposure variables [16]. Pearson's correlation test was utilized to determine the correlation between fetal outcome (gestational age at delivery, fetal weight, one-minute Apgar score) and maternal paraclinical parameters in both groups. Statistical significance was considered for results with p<0.05.

## Results

Out of 5775 births registered in the University Emergency Hospital in Bucharest during the period of our study, 145 patients were SARS-CoV2 positive, and only 13 were diagnosed with autoimmune thyroiditis with hypothyroidism and eligible for our research (Table 1). The prevalence of SARS-CoV2 infection associated with pregnancy was 2.51% over this period, while the prevalence of hypothyroidism in pregnant women with COVID-19 was 8.96%. All the pregnancies included in the study were singletons and no assisted reproduction techniques were documented.

**Table 1:** Distribution of patients based on SARS-CoV2 infection and hypothyroidism throughout the study period SARS-CoV2: severe acute respiratory syndrome coronavirus 2

Study years	Total number of pregnancies (n)	Total number of SARS-CoV2 pregnancies (n)	Total number of SARS-CoV2 pregnancies associated with hypothyroidism (n)
2020	2151	21	3
2021	2227	77	6
2022	1397	47	4

The maternal and fetal demographic and clinical features are described in Table 2.

**Table 2:** Demographic and clinical features of the groups under study *p <0.05 was considered statistically significant SARS-CoV-2: severe acute respiratory syndrome coronavirus 2; NICU: neonatal intensive care unit; COVID-19: coronavirus disease 2019

Demographic Data		COVID-19 Group	COVID-19 Associated With Hypothyroidism Group	p-value*
Maternal demographic data				
Maternal age (years), mean ± SD		29.25 ± 6.23	34.07 ± 5.18	0.011*
BMI (kg/m^2^), mean ± SD		20.21 ± 3.27	24.10 ± 2.83	0.197
Area of residence	Urban, n (%)	82 (62.12%)	10 (76.92%)	0.165
	Rural, n (%)	50 (37.88%)	3 (23.08%)	
Investigated pregnancy, n (%)		91 (68.94%)	12 (92.31%)	0.337
Days of hospitalization, mean ± SD		4.48 ± 4.52	3.38 ± 2.22	0.053
Thrombophilia, n (%)		8 (6.06%)	2 (15.38%)	0.168
Pregnancy-induced hypertension, n (%)		10 (7.57 %)	0	-
Gestational diabetes, n (%)		7 (5.30%)	2 (15.38%)	0.168
Genito-urinary infection, n (%)		31 (23.48%)	2 (15.38%)	0.168
Emergency cesarean section, n (%)		83 (62.87%)	12 (92.31%)	0.077
ICU admission, n (%)		8 (6.06%)	0	-
SARS-CoV-2 pneumonia, n (%)		6 (4.54%)	0	-
Fetal outcomes				
Birth weight (grams), mean ± SD		3011.44 ± 672.79	2984.62 ±478.58	0.465
1-minute Apgar score, mean ± SD		8.45 ± 1.65	8.67 ± 0.78	0.658
Intrauterine growth restriction, n (%)		41 (31.06%)	4 (30.76%)	0.168
NICU admission, n (%)		31(23.48%)	2 (15.38%)	0.168
Stillbirth, n (%)		0	1 (7.69%)	-
Gestational age (weeks), mean ± SD		37.75 ± 2.55	38.31 ± 1.11	0.152

There was a statistically significant correlation between the two groups regarding the maternal age. Maternal comorbidities such as pregnancy-induced hypertension, gestational diabetes, or genitourinary infections were predominantly observed in cases of pregnant women without hypothyroidism. Among the pregnant women requiring ICU support, two had gestational diabetes, while the remainder developed SARS-CoV-2 pneumonia and received remdesivir treatment. Only one patient with hypothyroidism needed remdesivir treatment (7.69%), a rate comparable to that of patients in the other subgroup, where 11 patients required this treatment (8.33%). This indicates that the presence of hypothyroidism does not entail a higher risk for antiviral treatment.

Despite the high percentage of emergency cesarean sections in both groups, the study did not find a positive correlation between this mode of delivery and SARS-CoV-2 infection. The main reasons for cesarean section were obstetrical causes, such as cephalopelvic disproportion or imminent uterine rupture.

While hypothyroidism is typically associated with a higher risk of preeclampsia, our study found 10 cases (6.89%) of induced-pregnancy hypertension in the total sample, yet none were observed in the group with thyroid dysfunction.

**Figure 1:**
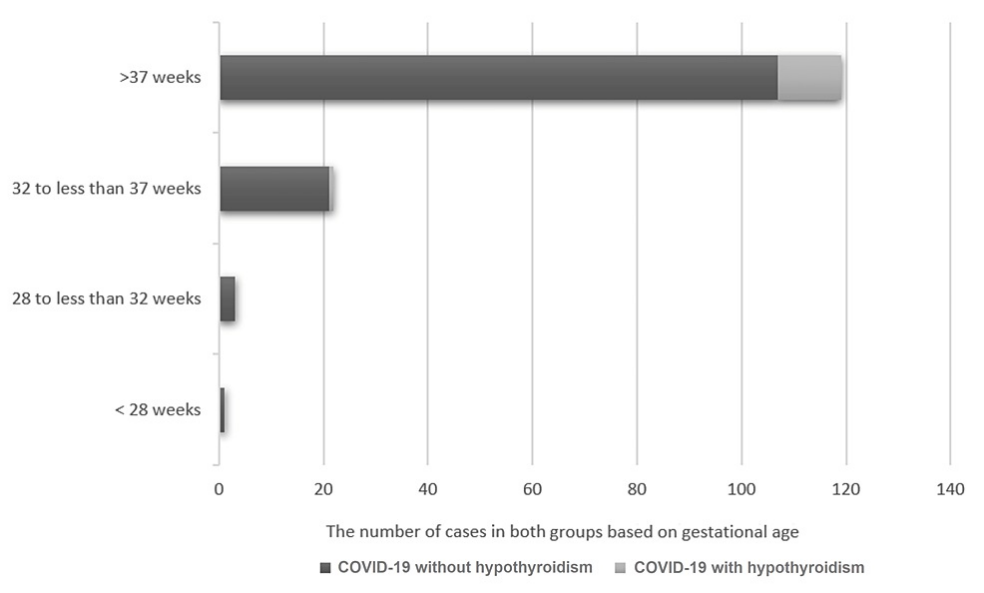
The distribution of preterm birth in the groups under study

Examining the risk of preterm birth, our investigation revealed that 36 patients (24.82%) across the entire study population experienced this complication. Within the group of patients diagnosed with both COVID-19 and hypothyroidism, preterm birth risk was observed in three cases (23.07%), with no cases of extremely preterm birth recorded (Figure 1).

**Figure 2:**
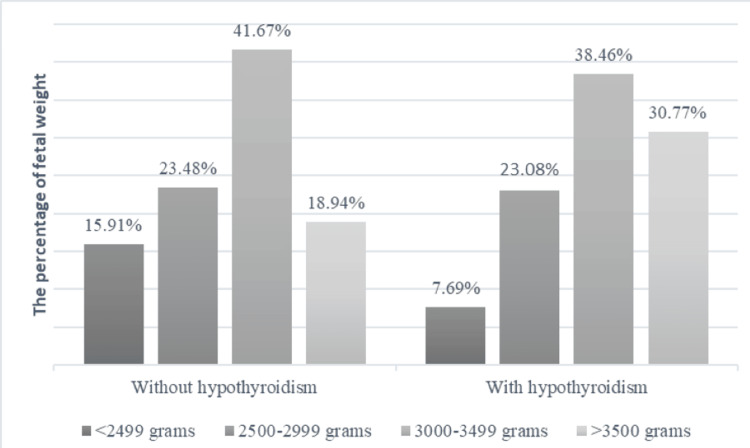
The distribution of fetal weight in the two groups under study

Considering fetal weight at birth, the mean average weight in the overall cohort was 3026.28 ± 659.54 grams. Within the group without hypothyroidism, we documented 21 neonates (15.91%) weighing less than 2499 grams, whereas in the other group, only one case with this weight (7.69%) was noted (Figure 2).

While there was no statistically significance regarding the one-minute Apgar score between the two groups, we revealed that in the group without hypothyroidism, there was a group of 16 neonates who developed respiratory distress (12.12%) with a mean one-minute Apgar score of 5.68 ± 0.13.

Intrauterine growth restriction was observed in four cases (30.76%) within the hypothyroidism group, a percentage similar to the 31.06% found in the other group, which had 41 cases. Concerning maternal blood count, we observed statistically significant differences between the two subgroups in terms of the value of INR (p=0.012) and uric acid value (p=0.013) (Table 3). In the entire group, patients requiring ICU support had a mean uric acid value of 6.74 ± 0.27 mg/dL, while those who did not require ICU support had a mean uric acid value of 4.93 ± 0.01 mg/dL. Moreover, the average number of hospitalization days for cases with high uric acid values was 15.38 ± 1.21, compared to 4.09 ± 0.02 in cases with lower uric acid values. Of the patients with hyperuricemia, 75% received remdesivir treatment.

**Table 3:** Maternal laboratory parameters in the groups under study *p <0.05 was considered statistically significant APTT: activated partial thromboplastin time; AST: aspartate aminotransferase; GOT: glutamic-oxalacetic transaminase; ALT: alanine transaminase; GPT: glutamate pyruva te transaminase; CRP: C-reactive protein; COVID-19: coronavirus disease 2019

Laboratory Parameters	COVID-19 Without Hypothyroidism Group, mean±SD	COVID-19 With Hypothyroidism Group, mean±SD	p-value*
Hemoglobin (g/dL)	11.10 ± 1.33	11.71 ± 1.34	0.468
Hematocrit (%)	33.27 ± 3.88	34.31 ± 3.59	0.943
Platelets (10^3^/uL)	230.22 ± 72.52	226.77 ± 52.73	0.476
Leukocytes (10^3^/uL)	11.26 ± 4.12	10.61 ± 4.53	0.964
APTT (second)	30.28 ± 4.7	29.72 ± 4.52	0.680
INR	1.05 ± 4.7	1.01 ± 0.11	0.012*
AST/GOT (U/L)	25.95 ± 0.16	26 ± 0.91	0.082
ALT/GPT (U/L)	21.67 ± 0.23	25.69 ± 1.76	0.095
Serum creatinine (mg/dL)	0.62 ± 0.41	0.56 ± 0.08	0.984
Serum urea (mg/dL)	21.46 ± 10.18	17.63 ±6.2	0.945
Uric acid (mg/dL)	9.44 ± 0.39	5.58 ± 0.07	0.013*
CRP (mg/dL)	3.53 ± 0.57	3.57 ± 0.73	0.472

Analyzing the one-minute Apgar score in the two groups, we found a statistically significant negative correlation between uric acid levels and fetal parameters (p<0.01), while in the group of pregnant women with COVID-19 alone, there was no correlation (p=0.814). Additionally, in both groups, there was a significant correlation between uric acid levels and the risk of intrauterine growth restriction (p=0.045 in the COVID-19 without hypothyroidism group and p<0.001 in the group of pregnant women with both COVID-19 and hypothyroidism).

While examining the C-reactive protein (CRP) value in the group with hypothyroidism, a mean value of 3.57 ± 0.73 was seen. Furthermore, a significant but negative correlation was found between the value of CRP and the value of TSH (p=0.007). No statistically significant correlation was established between the value of CRP and fT4 (p=0.752). The mean value of hemoglobin and hematocrit in the entire group was within the limits. No renal or hepatic cytolysis was observed in the subgroups under study.

Our study established prolonged INR values in the group with only COVID-19; in the cases with SARS-CoV-2 pneumonia, the mean value of INR was 1.31 ± 0.09 and none of the patients had thrombophilia. The mean value of INR in the hypothyroidism group was slightly lower (1.01 ± 0.11). Also, a statistically significant correlation was found between the values of INR and the one-minute Apgar score in both groups (p<0.001).

## Discussion

SARS-CoV-2 infection poses a life-threatening condition, exhibiting higher morbidity and mortality rates among pregnant women, who are regarded as a vulnerable group due to the immunosuppression associated with pregnancy [17-19]. A study performed by Zambrano et al. on 461,825 reproductive-age women with positive RT-PCR for SARS-CoV-2 infection showed an incidence of 6.6% in pregnant women. The increased incidence of SARS-CoV-2 infection during pregnancy was attributed to meticulous surveillance via RT-PCR within this demographic. According to the report by Zambrano et al., which utilized data from the Centers for Disease Control and Prevention (CDC) via national COVID-19 case surveillance and the National Notifiable Diseases Surveillance System (NNDSS), pregnant women infected with SARS-CoV-2, particularly those with underlying health conditions, were at increased risk for cardiorespiratory complications and complex symptomatology [4]. The current study showed an incidence rate of 2.51%, with 145 cases among 5775 pregnant women who delivered in our unit. Of these, 57 cases (39.31%) involved women aged 30-35 years. The number of pregnant women aged 18-29 years (56 cases) represented 38.62%, which is lower than the percentage reported in a study conducted by Virk et al. (57.3%) which included 48,445 third-trimester pregnant women [20]. 

Conversely, in the group with hypothyroidism, there was a significant difference in maternal age, which was higher (34.07 ± 5.18 years) compared to the group without hypothyroidism (29.25 ± 6.23 years). The association between COVID-19 and autoimmune diseases during pregnancy is elucidated by the heightened release of inflammatory cytokines and chemokines, occurring alongside a suppressed immune system [1,2,21]. Considering autoimmune thyroiditis with hypothyroidism, it is hypothesized that SARS-CoV-2 infection exacerbates the pro-inflammatory response in pregnant women with hypothyroidism, potentially resulting in adverse outcomes [22]. Although autoimmune thyroiditis with hypothyroidism has a prevalence of 2-3% during pregnancy [10], our study showed that pregnant women with hypothyroidism are predisposed to SARS-CoV-2 infection, with a prevalence of 8.96%.

Concerning adverse pregnancy outcomes such as placental insufficiency, it is established that hypothyroidism diminishes the proliferation of trophoblast cells and enhances apoptosis, as thyroid hormone plays a multifaceted role in maintaining normal placental function [23-26]. Additionally, hypothyroidism is characterized by an altered immune status leading to decreased levels of anti-inflammatory cytokines [27]. By proposing similar mechanisms, the overlap of SARS-CoV-2 infection on a background of hypothyroidism exacerbates pregnancy outcomes, elevating the risk of preterm birth or intrauterine growth restriction.

In the current study, the risk of preterm birth among the entire cohort of pregnant women positive for SARS-CoV-2 was lower (24.82%) compared to the findings reported by Bellos et al. in their 2021 research (29.7%) [14]. Nonetheless, the risk of preterm birth in pregnant women with COVID-19 remains higher than the global prevalence of preterm births (9.6%) [28]. Within our study, the group of pregnant women with hypothyroidism exhibited a preterm birth rate of only 7.69%, indicating that this condition did not exacerbate the risk of this pregnancy complication observed in the overall COVID-19 group.

In a meta-analysis conducted by El-Atawi et al. in 2024, a heightened risk for low birth weight in pregnancies affected by COVID-19 was revealed. Consequently, analyzing four studies with a random-effects model, the researchers found that neonates born to mothers who tested positive for COVID-19 had a significantly higher risk of low birth weight compared to those born to mothers without COVID-19 (OR = 1.86, 95%CI: 1.27-2.72, p = 0.001) [29]. Upon examining these findings, the average fetal weight observed in our study (3026.28 ± 659.54 grams) closely resembled that reported by Li et al. in 2021 (3066.7 ± 560.2 grams, with a difference of -250.40 grams compared to a cohort of non-COVID pregnant women) [30]. Their study showed that the risk of low birth weight was 17.6% in confirmed COVID-19 cases and 10.5% in suspected cases, compared to 2.5% in pregnant women without COVID-19. In our study, considering both groups, the risk of birth weight under 2500 grams was 15.17%, similar to the previously mentioned rates.

The current study found no statistically significant correlation between the concurrent presence of hypothyroidism and COVID-19 regarding fetal weight at birth, with a slight negative difference observed in the autoimmune thyroiditis subgroup (2984.62 ± 478.58 grams vs. 3011.44 ± 672.79 grams). Additionally, within the hypothyroidism group, only one case of fetal weight under 2500 grams was registered, involving a pregnant woman without any comorbidities. Furthermore, the presence of gestational diabetes or thrombophilia in pregnant women with both COVID-19 and hypothyroidism did not indicate a decrease in fetal weight (3030 ± 410.12 grams and 3235 ± 120.21 grams, respectively).

Our study found that the one-minute Apgar score, the risk of intrauterine growth restriction and NICU admission are not affected by the presence of hypothyroidism in pregnant women with COVID-19. However, an association between these conditions was observed in a case resulting in stillbirth. Fetal respiratory distress was associated with COVID-19 in 16 cases (12.12%), with surfactant administration needed in six of these cases (37.5%). However, none of these complications occurred in the hypothyroidism subgroup.

Regarding maternal complications, we observed that the presence of hypothyroidism did not exacerbate the course of COVID-19. In the group with COVID-19 and hypothyroidism, we did not record any cases of maternal ICU admission or SARS-CoV-2 pneumonia. The prevalence of pregnancy-induced hypertension in the COVID-19 group was 7.57%, similar to the rate reported by Karimi-Zarchi et al. in 2021 (8.2%) [31]. Although hypothyroidism is associated with a higher risk of preeclampsia [32], none of the patients in our study developed this complication, likely because levothyroxine treatment was initiated in every case prior to pregnancy.

Concerning paraclinical examinations, it is suggested that low baseline uric acid levels are linked to increased ICU admission in COVID-19 patients [33]. In our study, we observed hyperuricemia in pregnant women with SARS-CoV-2 (9.44 ± 0.39 mg/dL), with lower uric acid levels in those who required ICU support (6.74 ± 0.28 mg/dL). Additionally, in cases involving hypothyroidism, serum uric acid levels were significantly lower (p=0.013). A statistically significant but negative relationship was identified between INR values, with lower levels observed in the hypothyroidism group. In both cohorts, the mean INR values remained within normal limits. Among patients in the COVID-19 alone group, those requiring ICU support exhibited an INR value of 1.24 ± 0.06, while patients necessitating remdesivir treatment had a value of 1.02 ± 0.13. These findings align with those reported by Zinellu et al., who noted a correlation between prolonged INR and the severity of COVID-19 [34].

Autoimmune thyroiditis does not impact hematological parameters, renal function, or hepatic function in pregnant women with COVID-19. While no statistically significant correlation was found between CRP values in the groups under investigation, it was observed that a significant correlation existed between CRP and TSH values in the hypothyroidism group (p=0.007). This finding aligns with data presented in a study by Atalay et al., which reported a significant negative relationship between CRP levels and FT3 and TSH levels (p < 0.001, p = 0.005) [11].

Our study unveiled a higher proportion of cases necessitating emergency cesarean section in the hypothyroidism group, albeit without a statistically significant correlation (p=0.077). On the other hand, in the hypothyroidism group, the primary indication for emergency cesarean section was imminent uterine rupture, with acute fetal distress noted in only one case. Conversely, fetal distress accounted for 13.63% of indications for emergency birth in the other group. These findings suggest that hypothyroidism status did not exacerbate fetal distress in pregnancies affected by COVID-19.

Study limitations

The primary limitation of this study is the small sample size, which is attributed to the low prevalence of cases involving pregnant women with COVID-19 and hypothyroidism. Another limitation is the absence of additional laboratory analyses required to determine how hypothyroidism might influence the progression of SARS-CoV-2 infection, such as serum FT3 levels. Conversely, the levels of TSH, fT4, and FT3 could unveil other significant aspects in pregnant women with COVID-19 and undiagnosed hypothyroidism.

## Conclusions

The link between SARS-CoV-2 infection and hypothyroidism does not appear to increase the risk of preterm birth, intrauterine growth restriction, or low fetal weight at birth. However, it may be associated with a higher risk of stillbirth. Larger-scale studies are needed to determine if there is a direct correlation. Moreover, hypothyroidism in pregnant women with COVID-19 does not exacerbate the progression of the viral infection, as it is not linked to an increased need for ICU support and is associated with a shorter hospitalization period. Additionally, the high incidence of emergency cesarean section in pregnant women positive for COVID-19 with hypothyroidism seems to be independent of thyroid pathology. The presence of hypothyroidism in pregnant women with COVID-19 correlates with lower maternal uric acid levels and a slight decrease in INR values.
